# A Ru-Based Complex for Sustainable One-Pot Tandem Aerobic Oxidation-Knoevenagel Condensation Reactions

**DOI:** 10.3390/molecules29215114

**Published:** 2024-10-30

**Authors:** Wael A. A. Arafa, AbdElAziz A. Nayl, Ismail M. Ahmed, Ayman M. S. Youssef, Asmaa K. Mourad, Stefan Bräse

**Affiliations:** 1 Department of Chemistry, College of Science, Jouf University, Sakaka 72341, Aljouf, Saudi Arabia; aanayel@ju.edu.sa (A.A.N.); imibrahim@ju.edu.sa (I.M.A.); 2 Department of Chemistry, Faculty of Science, Fayoum University, Fayoum P.O. Box 63514, Egypt; ams12@fayoum.edu.eg (A.M.S.Y.); akk00@fayoum.edu.eg (A.K.M.); 3 Institute of Biological and Chemical Systems—Functional Molecular Systems (IBCS-FMS), Kaiserstrasse 12, 76131 Karlsruhe, Germany

**Keywords:** Ru-complex, aerobic oxidation, one-pot reaction, Knoevenagel condensation

## Abstract

Our novel binuclear complex-anchored Ru(III) catalyst, designed and assembled by sonicating 2,2′-(4,6-dihydroxy-1,3-phenylene)bis(1*H*-benzo[*d*]imidazole-4-carboxylic acid), Ru(DMSO)_4_Cl_2_ and 4-methylpyridine, demonstrates remarkable efficiency and selectivity. It promotes one-pot reactions, including active methylenes and benzyl alcohols in water, via a tandem aerobic oxidation/Knoevenagel condensation process, yielding benzylidene malononitrile in excellent yields. The catalyst’s ability to oxidize benzyl alcohols to aldehydes, which then undergo Knoevenagel condensation with active methylenes, makes it a multifunctional catalyst. Notably, the catalyst can be successfully retrieved and recycled for five successive rounds with no significant decrease in catalytic efficiency. The ICP study showed that no catalyst leaching was observed, indicating that the designed catalyst is indeed heterogeneous. The Ru catalyst outperformed other documented catalysts in terms of lower dose, shorter duration, decreased working temperature, and the absence of dangerous additives. This demonstrates the catalyst’s robustness and sustainability, making it a promising candidate for future organic conversions and industrial uses.

## 1. Introduction

Recently, one-pot reactions have received a lot of interest as an appealing synthetic strategy for enhancing overall process effectiveness and cutting down on waste and energy consumption during production [1,2,3,4,5]. One of the challenges facing one-pot conversion is designing heterogeneous catalysts with multiple catalytically active sites that could support several reactions independently and concurrently without interfering with one another [6,7,8]. α,β-unsaturated nitriles are essential building blocks for producing fine chemicals and drugs [9,10]. Such compounds have demonstrated incredible antitumor activities targeting key tumor cells, including Topo-I/II, microtubules, DNA, and kinases [11,12]. Furthermore, antioxidant and antimalarial properties have been demonstrated by arylidine derivatives [13]. These intermediates’ production can typically be developed by the base-catalyzed Knoevenagel condensations of ketones or aldehydes with active methylenes [14]. Nonetheless, the catalytic procedure faces restricted substrate diversity due to the substantial cost or inaccessibility of several aldehydes [14]. Alcohols are more readily available and less expensive than aldehydes, making them suitable as starting substances in such reactions. A powerful multifunctional catalyst is required when designing a heterogeneous promotor for the aerobic oxidation-Knoevenagel process. Long-established oxidation strategies for transforming alcohol to aldehyde in tandem processes typically comprise stoichiometric quantities of expensive and potentially harmful inorganic oxidants [10,15,16]. The development of different heterogeneous and homogeneous catalysts comprising noble metals, namely Pd, Au, Ir, and Pt, for the controlled aerobic oxidation of alcohols has also been reported [17,18,19,20]. Considering how costly other noble-metal catalysts are, Ru catalysts are more appealing economically. Ru(III) complexes have a long history of being effective oxidation catalysts for various reactants, including sulfides, alcohols, and aldehydes [21]. However, most investigations that utilize ruthenium, whether heterogeneously or homogeneously, employ non-environmentally friendly oxidants, such as iodosylbenzene and periodic acid [22,23]. Very few published studies currently discuss utilizing Ru-contained complexes in the aerobic oxidation of alcohols [24,25,26]. Furthermore, such catalysts frequently have intrinsic drawbacks, including the requirement for high catalytic loads (5.0 mol% [Ru]) or a considerable oversupply of O_2_ (20.0 atm) [27,28]. Therefore, the development of a more atom-effective and sustainable protocol that uses reusable catalysts and O_2_ as an oxidant is urgently needed. In recent decades, the utilization of ultrasound irradiation in multicomponent organic reactions has grown significantly [29]. This eco-friendly technique is a reasonably reliable method for recognizing how substance and energy interact to produce chemical and physical modifications. Ultrasound irradiation can enhance traditional reactions that require acidic or basic conditions, reagents, elevated temperatures, undesirable outcomes, and long reaction times [30]. Utilizing ultrasound irradiation, a liquid’s particles can effectively produce inflated bubbles. In subsequent compression rounds, the bubbles cave in, producing the energy needed for physicochemical changes. Therefore, ultrasound offers several benefits in organic reactions, including improved reaction rates, higher yields, selectivity, reduced hazardous substances, and simpler conditions, making it more practical and conveniently controlled when compared with conventional techniques [31]. In the present study, a binuclear complex-anchored Ru(III) was designed, synthesized, and characterized. The efficiency of the developed Ru catalyst was evaluated to promote one-pot tandem oxidation/Knoevenagel condensation involving active methylenes and benzyl alcohols under eco-friendly conditions. The catalyst effectively converted the alcohols in superior yields and selectivity into the intended Knoevenagel condensation products. Additionally, the catalyst showed excellent recyclability and stability, which made it a viable option for practical applications in organic transformations.

## 2. Results and Discussion

### 2.1. Assembly of Ru Catalyst (***4***)

**Scheme 1:**
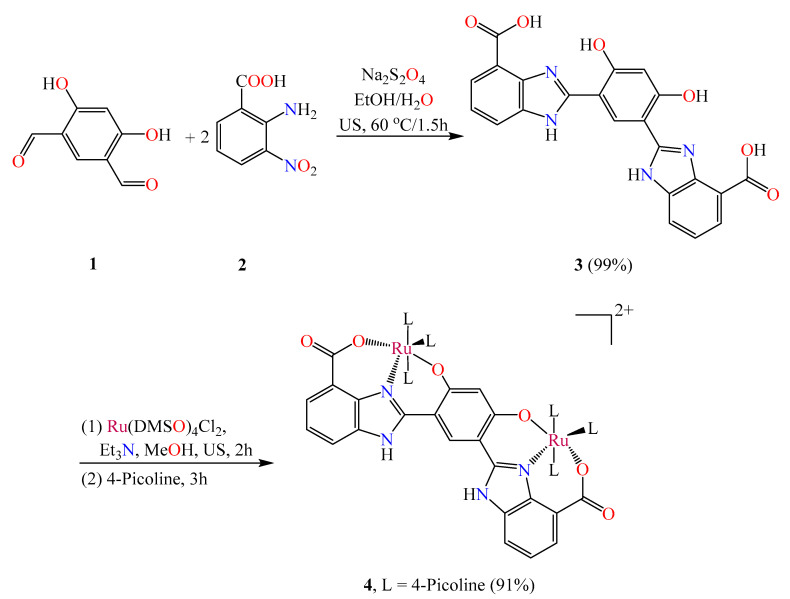
Synthesis of bis-benzimidazole derivative (**3**) and the corresponding Ru complex (**4**).

Scheme 1 displays the assembly approaches for the target ligand (**3**) and its binuclear Ru-complex (**4**). In the beginning, 2-amino-3-nitrobenzoic acid (**2**) and 4,6-dihydroxyisophthalaldehyde (**1**) underwent a reductive cyclization process, applying a reducing agent (Na_2_S_2_O_4_) to produce the ligand scaffold (**3**) through sonosynthesis. The outstanding yield (99%) of the bis-benzimidazole derivative **3** was achieved under the above conditions. The obtained derivative exhibited ESI-HRMS, NMR spectra, and an elemental analysis consistent with the proposed structure (SI, Figures S1–S3). The ^1^H NMR of the product (**3**) afforded two distinct singlets at 14.01 and 11.50 ppm assigned for the COO*H* and N*H* protons, respectively. Furthermore, detecting the signals at 166.7 and 141.0 ppm (^13^C NMR) revealed the presence of the carboxylic *C*=O and *C*=N motifs, respectively. The distinctive stretching band at 1709 cm^−1^ characteristic of the aldehydic carbonyl group vanished from the Fourier transform infrared (FTIR) spectrum of compound **3**, indicating the full uptake of the reacting substances. Furthermore, distinct C=O, O-H, and N-H bands at 1679 and 3290–3084 (broad) cm^−1^ confirmed the formation of bis-benzimidazole derivative **3**.

**Figure 1:**
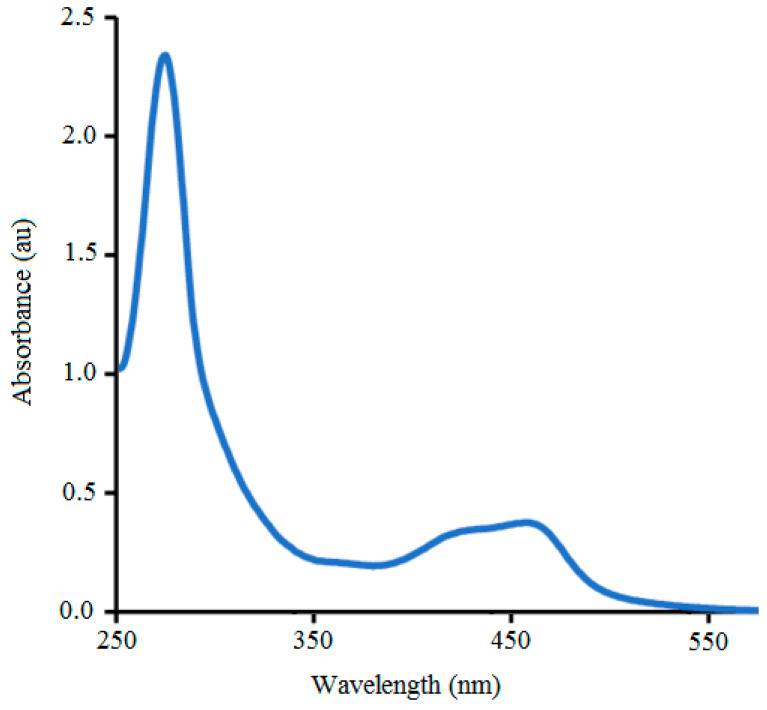
UV-Vis spectrum of Ru complex (**4**) at 298 K in ethanol.

Second, the following protocol was implemented to synthesize the Ru complex (**4**) appropriately. After ligand **3** and [Ru(DMSO)_4_Cl_2_] were sonicated in the existence of triethyl amine for over 2 h, 4-picoline was introduced, and the sonication was then carried out for an additional 3 h, affording complex **4** a 91% yield (Scheme 1). It was observed that the developed binuclear Ru complex (**4**) is insoluble in ethyl acetate, toluene, tetrahydrofuran, and water. In contrast, it is soluble in commonly used organic solvents, including acetonitrile, ethanol, and dimethylformamide. The chemical structure of the assembled complex (**4**) was identified employing FTIR, ^1^H NMR, and ESI-HRMS as well as an elemental analysis (SI, Figures S4 and S5). However, many endeavors for growing single crystals, such as slow evaporation, slow cooling, vapor diffusion, and solvent diffusion [32] derived from the developed complex (**4**) were unsuccessful. The synthesized organoruthenium complex (**4**) was then measured with a magnetic moment. The results showed that the reading obtained is 1.80 B.M. Based on these results, Ru(III) (single electron paramagnetic) is assigned to the intended Ru-complex (**4**). The ^1^H NMR spectrum of the obtained complex (**4**) displayed unresolved signals attributed to the paramagnetic character of Ru(III). Consequently, a reducing agent such as ascorbic acid was introduced to induce the reduction of Ru(III) (paramagnetic) to Ru(II) (diamagnetic) state. The equatorial 4-picoline proton chemical shifts appear further down-field in the complex’s ^1^H NMR than the axial protons indicating a stronger deshielding effect from the equatorial ligand than from the axial ligand. For example, compound **4** exhibited a singlet for methyl motif at 2.29 ppm and a pair of signals at around 6.81 and 8.40 ppm (aromatic protons) ascribed to the axial protons of 4-picoline. Meanwhile, the appearance of one singlet at 2.33 ppm (CH_3_) and two signals at about 7.02 and 8.52 ppm (aromatic protons) are attributed to the equatorial protons of 4-picoline. Furthermore, ESI-HRMS supported the predicted stoichiometries of complexes, affording a [M] ion for catalyst **4**. The elemental analysis of the Ru complex (**4**) is consistent with the estimated value, confirming the binuclear framework hypothesized for the Ru complex. We further investigate the coordination nature of the Ru ions with the ligand by comparing the FTIR spectra of both complex (**4**) and ligand (**3**). Ligand **3** exhibited a strong vibration band at 1609 cm^−1^, attributed to the imine motif, which decreased to 1593 cm^−1^ in the complexation process. This shift in the vibration band indicates that the azomethine nitrogen atom interacts with the metal center in complex **4**. This suggests that the coordination of the metal ion affects the electronic environment around the C=N bond of ligand **3**. The disappearance of the broadband attributed to the O-H (around 3197 cm^−1^) of the ligand and the augmentation in the stretching frequency of C-O (1231 cm^−1^) in the complex spectrum suggest that the ligand has lost its proton and Ru formed a coordination bond with the oxygen atom. This deprotonation indicates a change in the ligand’s chemical structure and confirms its involvement in the coordination process. Additionally, the FTIR of the complex showed distinctive bands at 475, 560, and 449 cm^−1^. These nonligand bands confirm the occurrence of Ru-Cl, Ru-O, and Ru-N bonds in the complex. The observed frequencies suggest a substantial coordination between ruthenium and oxygen or nitrogen atoms. The UV-Vis spectrum of the established complex also showed a couple of absorbing peaks: the band’s presence at around 465 nm is ascribed to the ruthenium complex’s ligand-to-metal charge transmission (LMCT). In comparison, the band at 279 nm can be related to ligand charge transmission (Figure 1).

### 2.2. Catalytic Performance

#### 2.2.1. Catalytic Performance of the Ru Catalyst in the Benzyl Alcohol Aerobic Oxidation Approach

It is widely recognized that the selectively catalyst-driven oxidation of alcohols to aldehydes is among the foremost significant reactions in present-day organic conversions and industries. Therefore, extensive research efforts have been dedicated to finding sustainable protocols and novel heterogeneous catalysts with improved activity, selectivity, and recyclability to meet the increasing demand for such fine chemicals. To assess complex **4**’s catalytic activity and improve the reaction conditions, the selective aerobic oxidation of phenylmethanol (**5a**) to phenylmethanal (**6a**) was launched as a template protocol. Several variables were investigated (Table 1), including energy sources, temperature, solvents, and catalyst/substrate proportions.

Benzyl alcohol (**5a**, 1.0 mmol) and Na_2_CO_3_ (0.3 equiv) in 5.0 mL of H_2_O were first heated to 100 °C without a catalyst. Notwithstanding, after boiling for 12 h under these conditions (Table 1, Entry 1), the anticipated benzaldehyde (**6a**) was not observed in the reaction mixture (TLC). Upon adding 0.1 mol% of catalyst **4** to the simulated reaction, a low yield (46%) of the intended aldehyde (**6a**) was produced (Table 1, Entry 2). By exposing the reaction to 80% intensity ultrasound irradiation (40 °C) for three hours, the conversion of benzyl alcohol (**5a**) to the desired benzaldehyde (**6a**) was further enhanced to 82% (Table 1, Entry 3). The efficiency of the catalyst was observed to be significantly reduced in solvents such as tetrahydrofuran, ethyl acetate, and toluene, resulting in a diminished yield of the desired product despite sonication for three hours (Table 1, Entries 4–6). Thus, we can infer that polar solvents have the ability to stabilize the charged intermediates in this reaction, thereby reducing the activation energy and accelerating the rate of reaction. Whereas, non-polar solvents are unable to stabilize the charged intermediates, resulting in higher activation energy and slower reaction rates compared to polar solvents. Alternative solvents like ethanol, acetonitrile, and dimethylformamide have not been utilized for such targeted aerobic oxidation to keep the reaction going under heterogeneous conditions. Regarding the ongoing catalytic process, water delivered the most satisfactory outcomes out of all the investigated solvents, confirming its solvent efficiency (Table 1, Entry 3). A range of operational temperatures were employed to enhance the productivity of the current ultrasound-assisted process. While the template reaction was first run at an ambient temperature (25 °C), the desired product (**6a**) was achieved in a low yield (Table 1, Entry 7). In just 120 min, the catalyst (**4**) successfully transformed the model substance into the intended product (**6a**) by raising the reaction temperature to 50 °C (Table 1, Entry 8). The reason behind this could be that the catalyst and substrate-containing bubbles that were created collapsed with sufficient energy to enhance the rate and yield of the reaction. Remarkably, after raising the temperature to 60 °C, the intended compound (**6a**) was produced in 90 min with a superior yield (95%) (Table 1, Entry 9). This result validates the hypothesis that the surface tension and viscosity of the reaction mixture diminish with increasing temperature, eventually resulting in the generation and collapse of the bubbles. Surprisingly, yield and reaction rates slightly reduce above 60 °C (Table 1, Entry 10). Higher temperatures promote the generation of extra bubbles, which ultimately restrict the waves’ ability to pass across the reaction medium and marginally affect the conversion process. After studying the time impact, it was found that 90 min was the appropriate duration (Table 1, Entry 9). Several bases were introduced to improve the catalyst’s efficiency even more (Table 1, Entries 11 & 12). In the existence of K_2_CO_3_ or Cs_2_CO_3_ (0.3 equiv), benzyl alcohol is catalytically converted to benzaldehyde at rates of 95% and 96%, respectively. By increasing the equivalents of Cs_2_CO_3_ to 0.5 equiv, the conversion rate improved to 99% (Table 1, Entry 13). Furthermore, the benzyl alcohol-benzaldehyde conversion was remarkably limited without Cs_2_CO_3_, suggesting that such a base additive is essential. Significantly, no over-oxidation end product (benzoic acid) was observed, demonstrating the Ru-catalyst’s excellent selectivity for benzaldehyde. Also, the transformation of phenyl methanol into benzaldehyde was not identified in the Ar atmosphere, indicating the necessity of oxygen as an oxidant (Table 1, Entry 14). The catalyst amount was also studied after identifying the proper temperature and time. A variety of catalyst doses (0.05, 0.2, and 0.5 mol%) were thus employed in the model reaction (Table 1). Anticipatedly, the number of active sites accessible for reaction increased with raising the mole percent of the catalyst from 0.05 to 0.1, resulting in improvements in both the rate of conversion and yield (Table 1, Entries 15 and 12). However, the process efficiency diminishes for catalyst loads above 0.1 mol% (Table 1, Entries 16 and 17). This might be an effect of the increased reaction viscosity brought on by a rise in the solid catalyst amount, which lessens the ultrasonic strength in certain reaction regions. Consequently, cavitation bubbles did not form optimally within these reactor sections. Consequentially, 0.1 mol% was determined to be the optimum catalytic molar ratio (Table 1, Entry 13). Although they are incapable of being reused, other catalysts, including RuCl_3_.H_2_O and Ru(DMSO)_4_Cl_2_ (Table 1, entries 18 & 19), are also able to catalyze the oxidation process but with low yields (43 and 37%, respectively). The catalytic efficiency of **4** was subsequently evaluated for the selective aerobic oxidation of assorted benzyl alcohol derivatives (Table 2). Table 2 shows that all varieties of benzyl alcohols exhibited superior performance in the oxidation process, providing the respective aldehydes with excellent yields. In addition, reaction duration and yield were not significantly impacted by the functional group’s nature. The Ru complex (**4**) presented herein could be superior for the heterogeneous selective aerobic oxidation of aryl methanol derivatives.

#### 2.2.2. Oxidation–Condensation Tandem Reaction

Along with aerobic oxidation, **4**’s catalytic activity for the tandem benzyl alcohol oxidation/Knoevenagel process was also studied. This reaction is a significant C–C coupling process frequently employed for fine chemicals manufacturing. The bifunctional (oxidation and condensation) Ru catalyst (**4**) was chosen, along with the reactants benzyl alcohol (**5a**) and malononitrile, to maximize the process conditions (Table 3).

An initial endeavor was carried out in the model reaction, which included water acting as the solvent, the Ru complex (**4**) serving as a promoter, and oxygen gas operating as the green oxidant. The reaction produces the desired 2-benzylidenemalononitrile (**7a**) in a high yield with high selectivity. Furthermore, the screening of the reaction mixture revealed no benzaldehyde or benzoic acid was formed in the end product. The tendency for activity is almost comparable to that of the oxidation process, possibly due to the considerably rapid progress of condensation as compared to oxidation. Also, no benzaldehyde was observed in any of the steps, suggesting that oxidation is the rate-determining step. Additionally, dehydration processes in water have been documented [33,34] with outstanding productivity when hydrophobic catalysts are employed. Further, the approach is more sustainable when using a water-based solution because it reduces hazardous pollutants. No product (**7a**), comprising benzyl alcohol and malononitrile, was detected in the tandem process when it was investigated without a catalyst. To broaden the potential application of the present protocol for the selective tandem alcohol oxidation-Knoevenagel process, catalyst **4** was evaluated using the optimal conditions for various benzyl alcohols in water (Table 3). According to the results displayed in Table 3, the final product yields (**7a–h**) and reaction time were not significantly impacted by the substituents attached to the benzyl alcohols. Additionally, superior yields of the related α,β-unsaturated nitriles (**7k** & **7l**) were provided by heteroaromatic alcohols, including thiophen-2-ylmethanol and furan-2-ylmethanol (Table 3). In addition, *trans*-cinnamyl alcohol was assessed, and a moderate product of derivative **7m** was received (Table 3). This is probably because the hydroxyl group has a low electron density since it is located comparatively away from the aromatic ring. Also, by using optimum conditions, an aliphatic alcohol like cyclohexane methyl alcohol was appropriate with the malononitrile nucleophile, even though the reaction rate was sluggish, and a low yield (**7n**, 23%) was obtained after 6 h of reaction duration (Table 3). Such poor yield might be due to the fact that the oxidation of aliphatic alcohols is significantly harder than the oxidation of benzylic alcohols because aliphatic alcohols are less reactive [35,36]. Remarkably, diols like (1*H*-pyrazole-3,5-diyl)dimethanol and 1,4-phenylenedimethanol were also part of the current catalytic oxidation-condensation reactions. Consequently, sonicating such diols (1.0 mmol) with malononitrile (2.0 mmol) under optimal reaction conditions resulted in the production of the respective bis(methanylylidene) malononitrile (**7o** & **7p**) in high yields (Table 3). Under the conditions of ultrasonic irradiations, the benzyl alcohol similarly reacted with different active methylene compounds, such as ethyl cyanoacetate or diethyl malonate, employing **4** as a heterogeneous catalyst. However, the behavior varied depending on the type of active methylene. For example, the reaction comprising phenyl methanol and ethyl cyanoacetate took place effectively, yielding a moderate amount of ethyl (benzylidene)cyanoacetate (**7p**, 68%, Table 3). Instead, the reaction involving diethyl malonate and benzyl alcohol yielded a low yield (**7r**, 36%, Table 3). The reactivity of methylene derivatives can be explained by their varying acidity, which decreases from malononitrile (pKa = 11.1) to ethyl cyanoacetate (pKa = 13.1) to diethyl malonate (pKa = 16.4). Diethyl malonate has a higher pKa (16.4), making it less likely to undergo deprotonation and limiting the Knoevenagel condensation reaction with benzaldehyde. This is due to the deprotonation of the active methylene motif, which is considered the initial step in Knoevenagel condensation. According to Table 3, the Ru catalyst (**4**) is recognized to be extremely effective in the tandem aerobic oxidation-Knoevenagel condensation process under mild reaction conditions. A significant conversion was achieved for all substrates within 120 min and at a low temperature of 60 °C. Additionally, complete selectivity was detected for the obtained products, suggesting that Ru catalyst (**4**) could be employed for the selective tandem oxidation-Knoevenagel condensation of different benzyl alcohols.

#### 2.2.3. The Recycling of Ru Catalyst **4**

**Figure 2:**
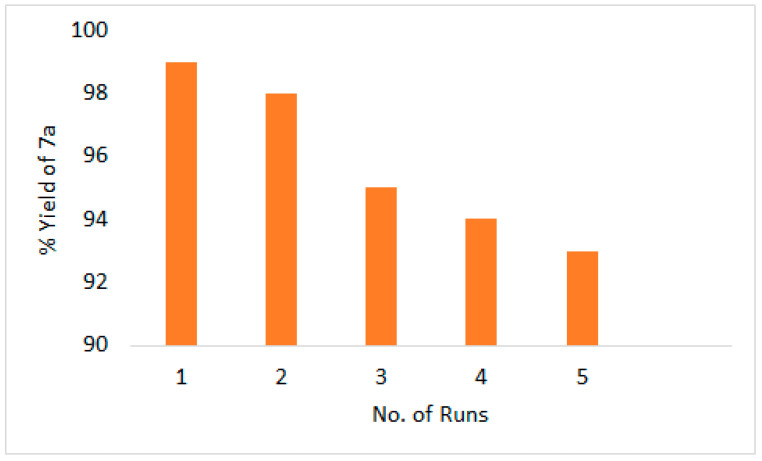
Reusability experiment of Ru catalyst (**4**) for assembly derivative **7a**.

The Ru catalyst (**4**) was evaluated for its potential for recycling the tandem aerobic oxidation-Knoevenagel condensation process involving malononitrile and benzyl alcohol. Once the reaction was completed, the α,β-unsaturated nitrile that was produced is dissolved in ethyl acetate. Following this, the paramagnetic catalyst **4** was separated by filtration, rinsed using ethyl acetate, dried under reduced pressure, and used again in the subsequent cycle employing identical conditions. Across the five recycling runs, outcomes ranging from 99% to 93% were achieved, exhibiting superior activity (Figure 2). The FTIR and ^1^H NMR spectra of the original and recovered catalyst appear quite similar, indicating that this catalyst is chemically stable and heterogeneous under optimal circumstances. Furthermore, even after five catalytic cycles, the ICP test demonstrated that the Ru percent in the catalyst (**4**) remains constant at 16.02%. The results above support the hypothesis that the structure remains unchanged throughout the catalytic runs, confirming the stability of the Ru complex for such tandem reactions.

#### 2.2.4. Test for Heterogeneity of Ru Complex (**4**)

**Figure 3:**
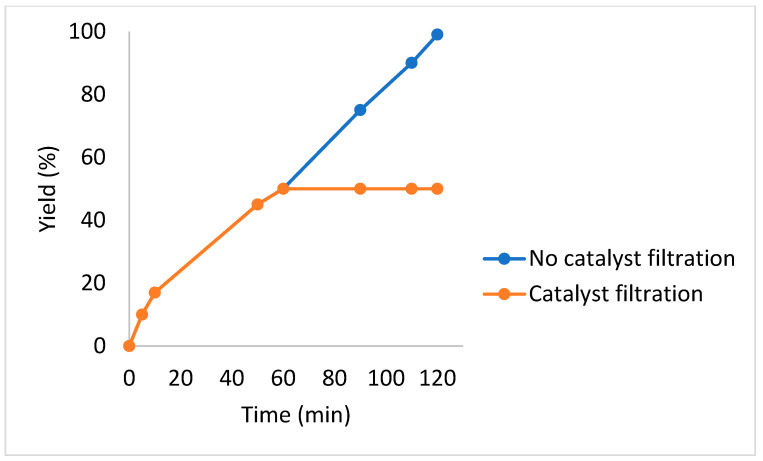
Benzyl alcohol transformation to derivative **7a** as a function of time employing catalyst **4**.

To investigate the potential for Ru catalyst leaching while carrying out the oxidation-Knoevenagel condensation reaction, a hot filtration experiment was conducted, and the catalytic process was halted after one hour. After that, the reaction mixture was centrifuged, and the leftover liquid was subjected to another hour of sonication. The result demonstrates that the transformation of benzyl alcohol ceased upon the removal of the catalyst (Figure 3). Thus, the possibility of partially heterogeneous/homogeneous catalysis was ruled out by this result. This finding highlights the benefit of the Ru’s stable covalently bound structure and that the Ru was not leaked, making it a prospective candidate for diverse uses. Furthermore, an ICP investigation was used to verify the heterogeneity of the Ru complex (**4**). Thus, following the catalyst’s removal, water was evaporated, and the leftover residue was dissolved in 5% NHO_3_. After employing ICP for assessing the acidic solution, it was found that there was a very limited amount of catalyst leaching (<0.20 ppm).

**Figure 4:**
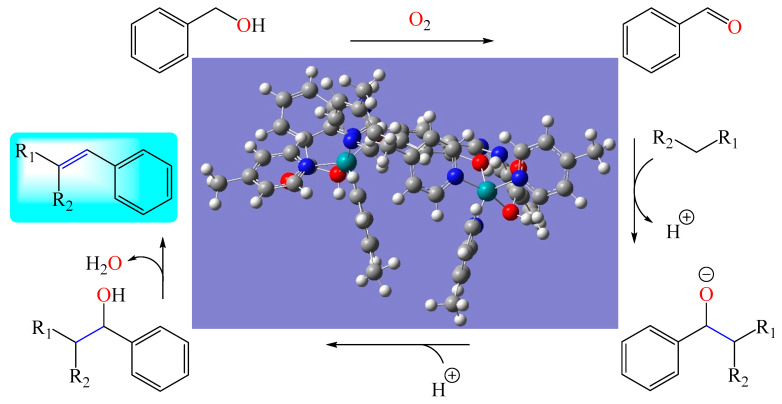
A suggested mechanism for the one-pot multicomponent reaction.

Figure 4 displays the proposed mechanism for the conversion comprising benzyl alcohol and active methylenes. Coupled with a Ru catalyst and basic Cs_2_CO_3_, the reaction commences with the aerobic oxidation of phenyl methanol to phenyl methanal. The Knoevenagel condensation process begins when the active methylene motif undergoes deprotonation, forming a carbanion intermediate. The generated carbanion underwent a nucleophilic attack on the benzaldehyde’s carbonyl moiety, yielding an additional intermediate that, after water removal, converted to benzylidene malononitrile (Figure 4).

Finally, the catalytic efficacy of the present protocol is compared with a variety of approaches that have been utilized to induce such a reaction (Table 4). The comparison displayed in Table 4 unambiguously demonstrates the superiority of the current protocol owing to its catalyst reusability, lack of necessitated large catalyst loading, use of O_2_ as a green oxidant, use of water as a solvent, employing of ultrasound energy, and easy reaction setup.

## 3. Experimental

### 3.1. General

The FTIR analysis employed a Fourier transform infrared spectrometer (Shimadzu IR-Tracer 100, Kyoto, Japan). An Agilent spectrophotometer (Agilent Technologies, Santa Clara, CA, USA) was utilized to record the UV-Vis absorption spectra. A Bruker 600 MHz NMR analyzer (Bruker Biospin GmbH, Ettlingen, Germany) monitored ^1^H and ^13^C NMR spectra. The content of Ru was estimated using an ICP-OES Optima 8000 atomic emission spectrometer (ThermoFisher Scientific, Dreieich, Germany). All of the substances were received from Sigma-Aldrich (St. Louis, MO, USA) and utilized without additional purification.

### 3.2. Synthesis of 2,2′-(4,6-Dihydroxy-1,3-phenylene)bis(1H-benzo[d]imidazole-4-carboxylic Acid) (***3***)

A mixture of 2-amino-3-nitrobenzoic acid (**2**, 2.0 mmol), 4,6-dihydroxyisophthalaldehyde (**1**, 1.0 mmol), and sodium dithionite solution (6.0 mmol, 1.23 g in 10.0 mL H_2_O), in ethanol (50.0 mL) was sonicated for 1.5 h at 60 °C. The formed yellow crystals (99%) were separated by filtration, washed with water (3 × 15.0 mL) and then acetone (3 × 15.0 mL), and dried. IR (KBr, cm^−1^): 3290–3084 (O-H + NH), 1679 (C=O), 1609 (C=N), 1219 (C–O). ^1^H NMR (400 MHz, DMSO-*d*_6_): *δ* 14.01 (s, 2H, 2COO*H*), 11.50 (s, 2H, 2N*H*), 7.88 (d, *J* = 8.5 Hz, 2H, Ar-H), 7.76 (d, *J* = 7.9 Hz, 2H, Ar-H), 7.45 (s, 1H, Ar-H), 7.28 (t, *J* = 7.6 Hz, 2H, Ar-H), 6.74 ppm (s, 1H, Ar-H). ^13^C NMR (100 MHz, DMSO-*d*_6_): *δ* 166.7, 151.4, 141.0, 135.2, 133.9, 132.0, 124.6, 122.6, 122.0, 117.4, 115.4, 106.7 ppm. ESI-HRMS *m/z* calcd. for C_22_H_14_N_4_NaO_6_ [M + Na^+^]: 453.0811; found 453.0815. Elemental analysis (%), calculated for C_22_H_14_N_4_O_6_: C, 61.40; H, 3.28; N, 13.02%; found: C, 61.49; H, 3.21; N, 12.97%.

### 3.3. Synthesis of Ru Complex (***4***)

A solution containing ligand **3** (0.5 mmol) and Et_3_N (8.6 mmol, 1.2 mL) [Ru(DMSO)_4_Cl_2_] (1.0 mmol, 484 mg) in methanol (10.0 mL) was sonicated for 2 h at 50 °C. After that, 4-picoline (24.0 mmol, 2.4 mL) was added, and sonication was continued for 3 h at 50 °C. The reaction was then terminated by pouring water (10.0 mL), and the isolated product was isolated by centrifugation, rinsed with diethyl ether (3 × 5.0 mL), and dried to give red crystals. The solid was purified using computerized flash chromatography (acetonitrile/methanol eluent and a 0% to 10% methanol gradient). The required product portions were combined, and the solvent was evaporated to afford complex **4** in 91% yield. IR (KBr, cm^−1^): 3128 (N-H), 1675 (C=O), 1593 (C=N), 1231 (C–O), 560 (Ru–O), 449 (Ru–N), 475 (Ru–Cl). ^1^H NMR (400 MHz, CDCl_3_ + 1.5 equiv ascorbic acid): *δ* 8.51–8.53 (4H, Ar-H), 8.39–8.42 (8H, Ar-H), 7.73–7.77 (4H, Ar-H), 7.60 (1H, Ar-H), 7.38–7.39 (2H, Ar-H), 7.03–7.03 (4H, Ar-H), 6.79–6.83 (8H, Ar-H), 6.62 (1H, Ar-H), 2.33 (6H, 2C*H*_3_), 2.29 (12H, 4C*H*_3_). HRMS (ESI) Calcd. for C_58_H_52_N_10_O_6_Ru_2_ [M^+^]: 1188.2158; found: 1188.2169. Elemental analysis (%), calculated for C_58_H_52_Cl_2_N_10_O_6_Ru_2_ ([M]Cl_2_): C, 55.37; H, 4.17; N, 11.13; Ru, 16.07%; found: C, 55.30; H, 4.21; N, 11.08; Ru, 16.02%. Uv-Vis (λ, nm): 279 and 465.

### 3.4. General Procedure for One-Pot Tandem Oxidation/Knoevenagel Condensation

In a round flask, water (5.0 mL), catalyst (0.1 mol%), benzyl alcohols (1.0 mmol), malononitrile (1.0 mmol), and Cs_2_CO_3_ (0.5 equiv) were added. A balloon filled with oxygen was attached to the vessel. The mixture was then sonicated at 60 °C, and the reaction progress was checked by TLC (ethyl acetate/*n*-hexane). The formed solid was extracted with ethyl acetate to afford a pure product. In the recyclability experiment, the Ru complex was separated by filtration, washed with ethyl acetate, and dried for 2 h under reduced pressure at 100 °C. The derivatives’ chemical structures were determined *by* matching their spectral analysis to authentic samples.

## 4. Conclusions

In summary, an effective heterogeneous catalyst was successfully assembled using a reductive cyclization process that included 2-amino-3-nitrobenzoic acid and 4,6-dihydroxyisophthalaldehyde, followed by complexation of the formed ligand with Ru(III). The anticipated structures of the established ligand and complex have been confirmed by employing FTIR, HRMS, and NMR techniques. The obtained Ru complex possesses distinctive bifunctional heterogeneous catalytic behavior and effectively catalyzed one-pot aerobic oxidation-Knoevenagel reactions. Moreover, the catalyst demonstrated a significant transformation of various benzyl alcohols with superior selectivity to the related benzylidene malononitrile utilizing O_2_ as an oxidant. The ICP and hot filtration experiment results also confirmed that the catalyst did not leach throughout the reaction. Further, the Ru catalyst can be reused for up to five cycles without significantly reducing its activity. Consequently, the Ru catalyst exhibited considerable stability, making it a bifunctional and cost-effective candidate for various organic conversions and manufacturing applications.

## Figures and Tables

**Table 1:** The behaviors of different catalysts in phenylmethanol oxidation ^a^.

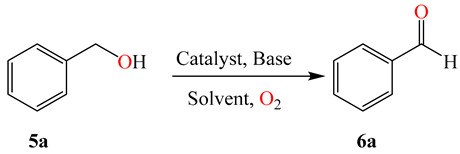							
Entry	Method	Catalyst (mol%)	Base	Solvent	Temp. (°C)	Time (h)	Yield (%)
1.	Silent	no	Na_2_CO_3_	H_2_O	100	12	ND
2.	Silent	**4** (0.1 mol%)	Na_2_CO_3_	H_2_O	100	12	46
3.	US	**4** (0.1 mol%)	Na_2_CO_3_	H_2_O	40	3	82
4.	US	**4** (0.1 mol%)	Na_2_CO_3_	Toluene	40	3	58
5.	US	**4** (0.1 mol%)	Na_2_CO_3_	Ethyl acetate	40	3	50
6.	US	**4** (0.1 mol%)	Na_2_CO_3_	THF	40	3	55
7.	US	**4** (0.1 mol%)	Na_2_CO_3_	H_2_O	rt	3	37
8.	US	**4** (0.1 mol%)	Na_2_CO_3_	H_2_O	50	2	92
9.	US	**4** (0.1 mol%)	Na_2_CO_3_	H_2_O	60	1.5	95
10.	US	**4** (0.1 mol%)	Na_2_CO_3_	H_2_O	70	1.5	93
11.	US	**4** (0.1 mol%)	K_2_CO_3_	H_2_O	60	1.5	95
12.	US	**4** (0.1 mol%)	Cs_2_CO_3_	H_2_O	60	1.5	96
13.	US	**4** (0.1 mol%)	Cs_2_CO_3_	H_2_O	60	1.5	99 ^b^
14.	US	**4** (0.1 mol%)	Cs_2_CO_3_	H_2_O	60	1.5	ND ^c^
15.	US	**4** (0.05 mol%)	Cs_2_CO_3_	H_2_O	60	1.5	88
16.	US	**4** (0.2 mol%)	Cs_2_CO_3_	H_2_O	60	1.5	97
17.	US	**4** (0.5 mol%)	Cs_2_CO_3_	H_2_O	60	1.5	95
18.	US	RuCl_3_.H_2_O	Cs_2_CO_3_	H_2_O	60	1.5	43
19.	US	Ru(DMSO)_4_Cl_2_	Cs_2_CO_3_	H_2_O	60	1.5	37

^a^ Reaction conditions: catalyst, benzyl alcohol (1.0 mmol), stirring in 5.0 mL solvent employing 1.0 atm O_2_ balloon. ND = Not Detected. ^b^ 0.5 equiv of Cs_2_CO_3_. ^c^ Under Ar atmosphere.

**Table 2:** Catalytic behavior of Ru catalyst (**4**) in aerobic oxidation approaches ^a^.

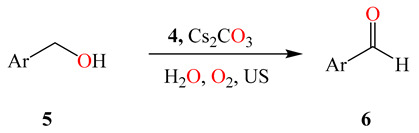
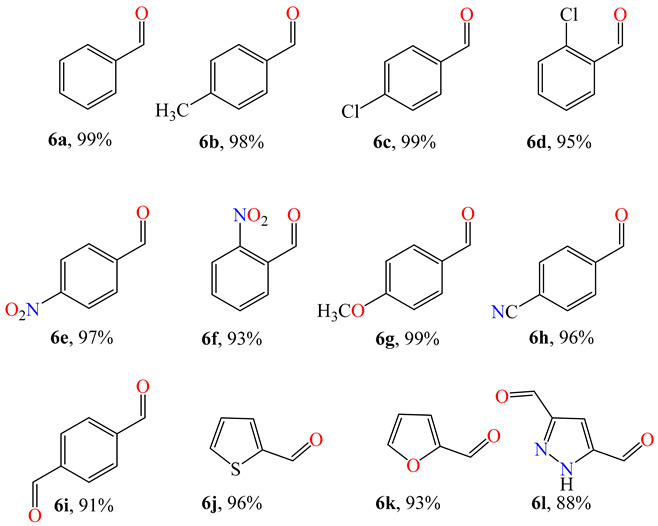

^a^ Reaction conditions: The catalyst (4, 0.1 mol%), benzyl alcohols (1.0 mmol), and Cs_2_CO_3_ (0.5 equiv) were sonicated in 5.0 mL water at 60 °C for 90 min employing a 1.0 atm O_2_ balloon.

**Table 3:** Catalytic behavior of Ru catalyst in tandem aerobic oxidation-Knoevenagel condensation reaction ^a^.

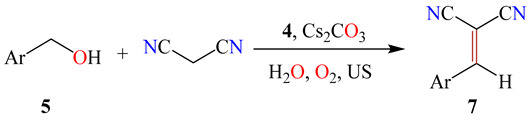
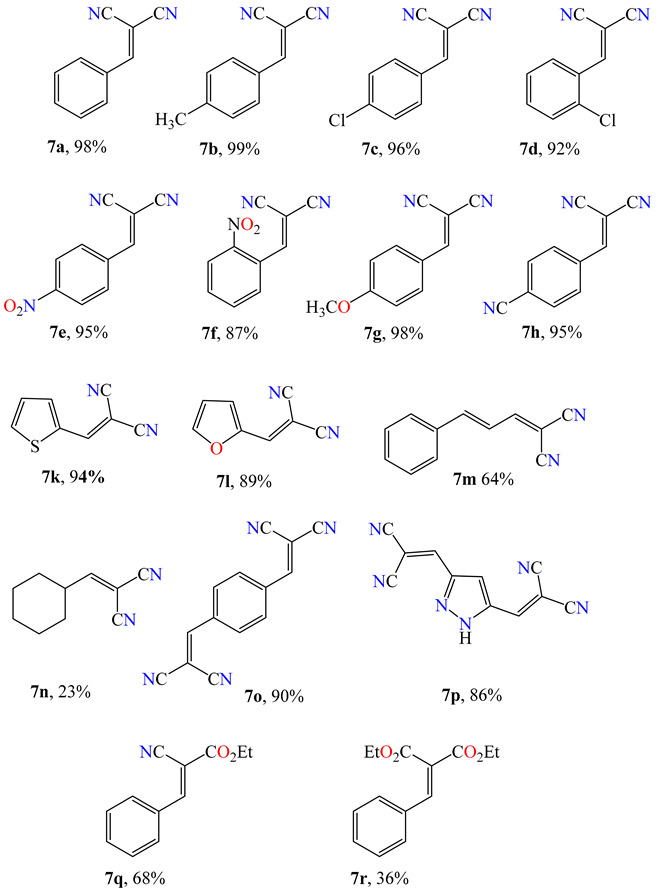

^a^ Reaction conditions: catalyst (**4**, 0.1 mol%, 0.2 mol% for **7o** & **7p**), benzyl alcohols (1.0 mmol), malononitrile (1.0 mmol, 2.0 mmol for **7o** & **7p**), and Cs_2_CO_3_ (0.5 equiv, 1.0 equiv for **7o** & **7p**) were sonicated in 5.0 mL water at 60 °C for 120 min employing 1.0 atm O_2_ balloon.

**Table 4:** Catalytic behavior of various catalysts for benzyl alcohol oxidation-condensation reactions.

Entry	Catalyst	Time (h)	Yield (%)	Ref.
1.	UiO-66-Ru	6 *	89.3	[37]
2.	Porph-UOZ-COF	60	93	[38]
3.	CsCu_2_I_3_@PCN-222(Fe)	15	96	[39]
4.	Ru^III^@bipy-CTF	13 *	99	[40]
5.	Fe_3_O_4_@SiO_2_@PEI@Ru(OH)x	22 *	90.2	[41]
6.	PorphCat-Fe	24	91	[42]
7.	CsCu_2_I_3_@PCN-222(Fe)	15	96	[39]
8.	PMoV_2_@DETA-MIL-101	36 *	99	[43]
9.	Pd/COF-TaPa-Py	5.5 *	98	[44]
10.	CoOx/tri-g-C_3_N_4_	6	96.5	[45]
11.	Ru catalyst (**4**)	1.5	99	This work

* The reaction occurred in two separate steps.

## Data Availability

Data are contained within the article and Supplementary Materials.

## Supplementary Materials

The following supporting information can be downloaded at: https://www.mdpi.com/article/10.3390/molecules29215114/s1, Figure S1: HRMS of ligand (**3**); Figure S2: ^1^H NMR (DMSO-*_d6_*) of ligand (**3**); Figure S3: ^13^C NMR (DMSO-*_d6_*) of ligand (**3**); Figure S4: ^1^H NMR (CDCl_3_) of Ru complex (**4**); Figure S5: HRMS of Ru complex (**4**).
