# Supplementary material for: A Ru-Based Complex for Sustainable One-Pot Tandem Aerobic Oxidation-Knoevenagel Condensation Reactions
Source: Molecules. 2024 Oct 30;29(21):5114. doi: 10.3390/molecules29215114 (PMC11547704; doi:10.3390/molecules29215114)
Supplement: Supplementary file 1 [file molecules-29-05114-s001.zip › molecules-3283826-supplementary.pdf]

Electronic Supporting Information

A Ru-Based Complex for Sustainable One-Pot Tandem Aerobic Oxidation-Knoevenagel Condensation Reactions

Wael A. A. Arafa*[1], AbdElAziz A. Nayl[1], Ismail M. Ahmed[1], Ayman M. S. Youssef[2], Asmaa K. Mourad[2], Stefan Bräse*[3]

[1]Department of Chemistry, College of Science, Jouf University, Sakaka 72341, Aljouf, Saudi Arabia

[2]Department of Chemistry, Faculty of Science, Fayoum University, P.O. Box 63514, Fayoum, Egypt

[3]Institute of Biological and Chemical Systems – Functional Molecular Systems (IBCS-FMS), Kaiserstrasse 12, 76131 Karlsruhe, Germany

*Authors to whom correspondence should be addressed.

Table of Contents

No.	Content	Page No.
1	Copies of NMR and HRMS	S2– S6

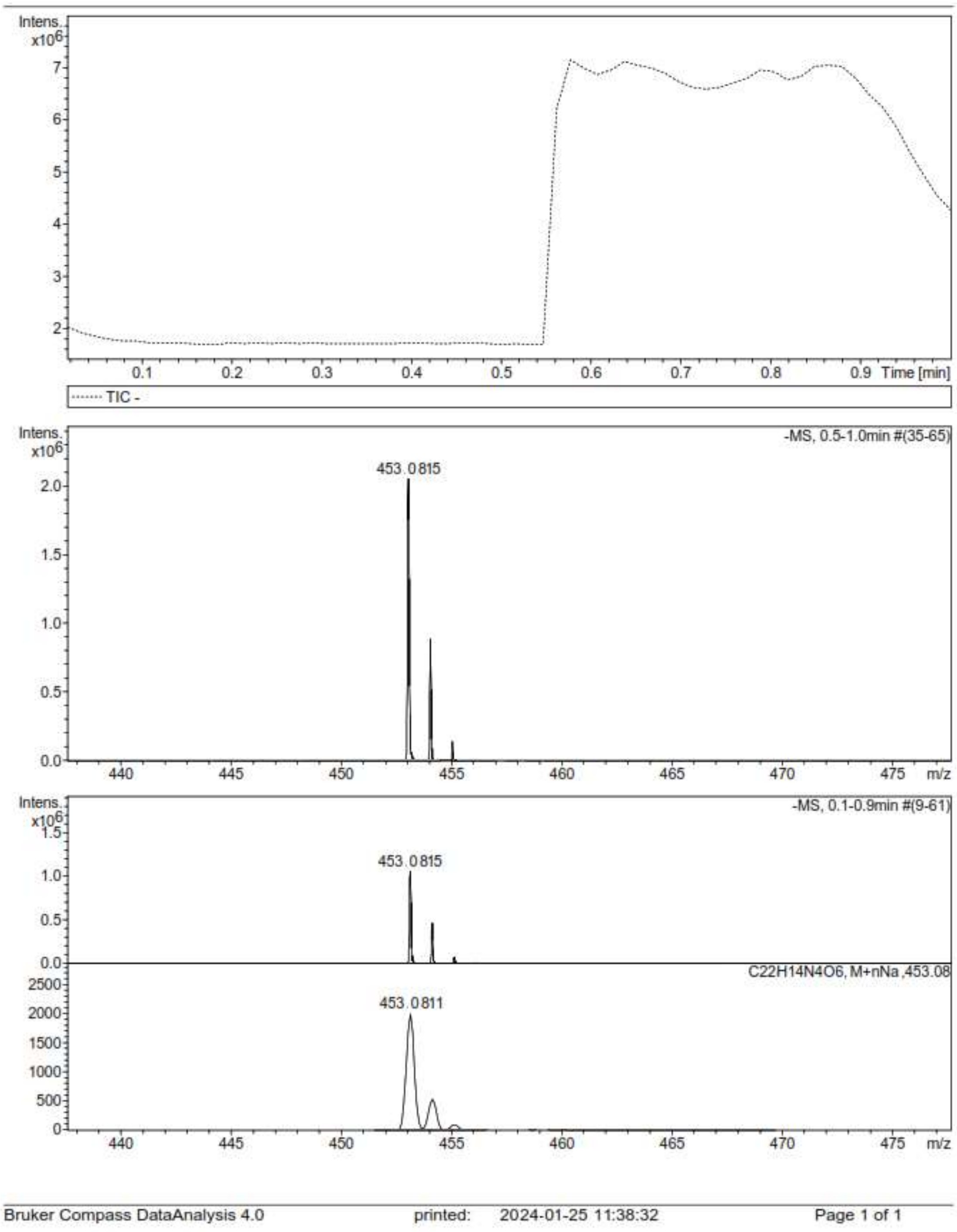

Fig. S1. HRMS of ligand (**3**)

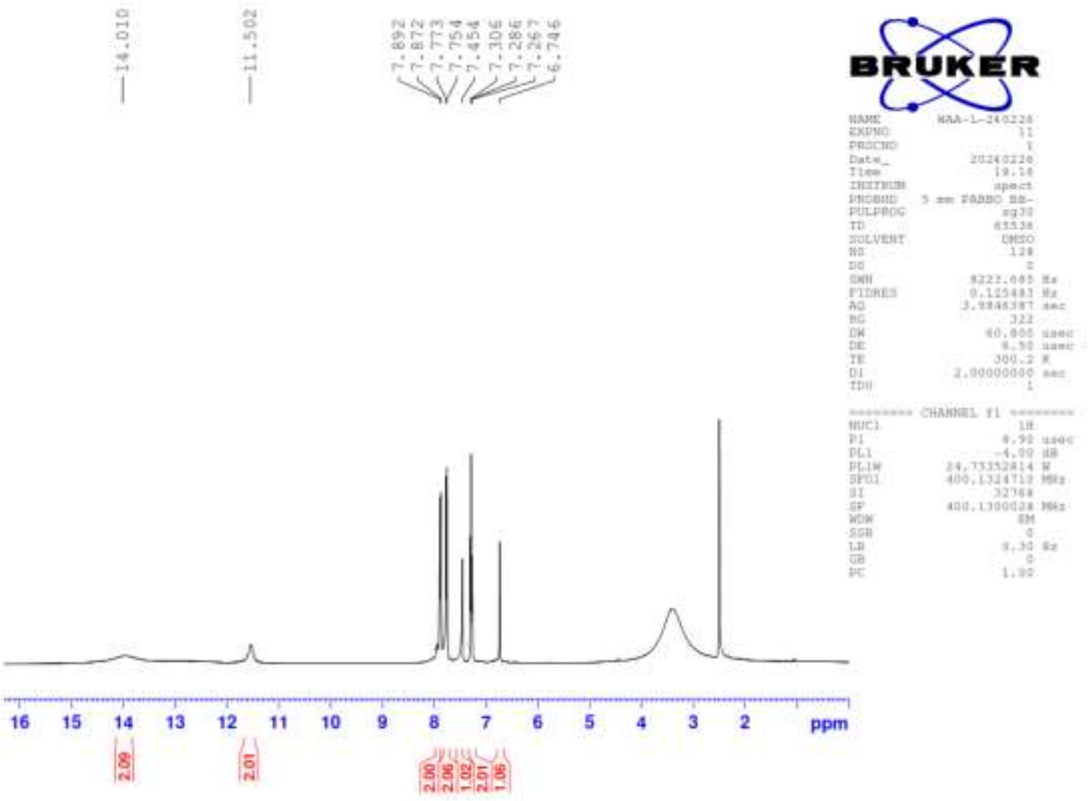

Fig. S2. ^{1}H NMR (DMSO-*d6*) of ligand (**3**)

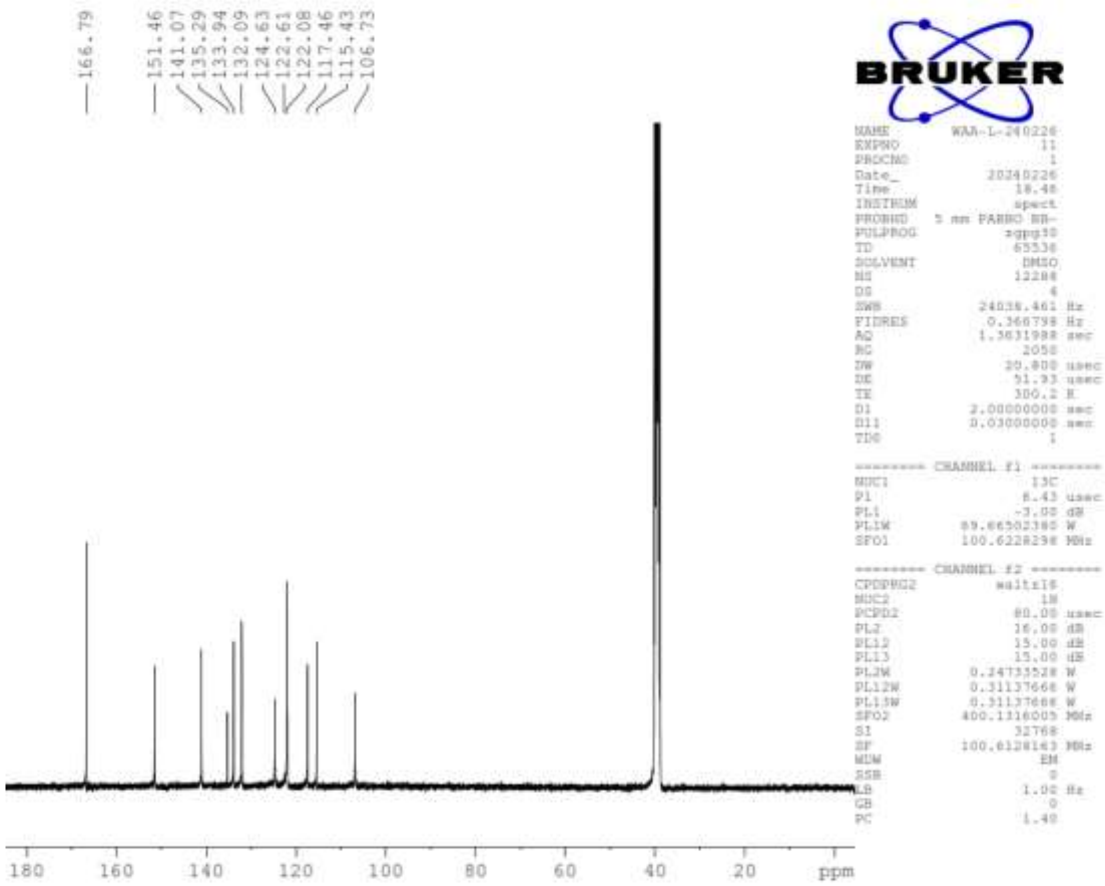

Fig. S3. ^{13}C NMR (DMSO-*d6*) of ligand (**3**)

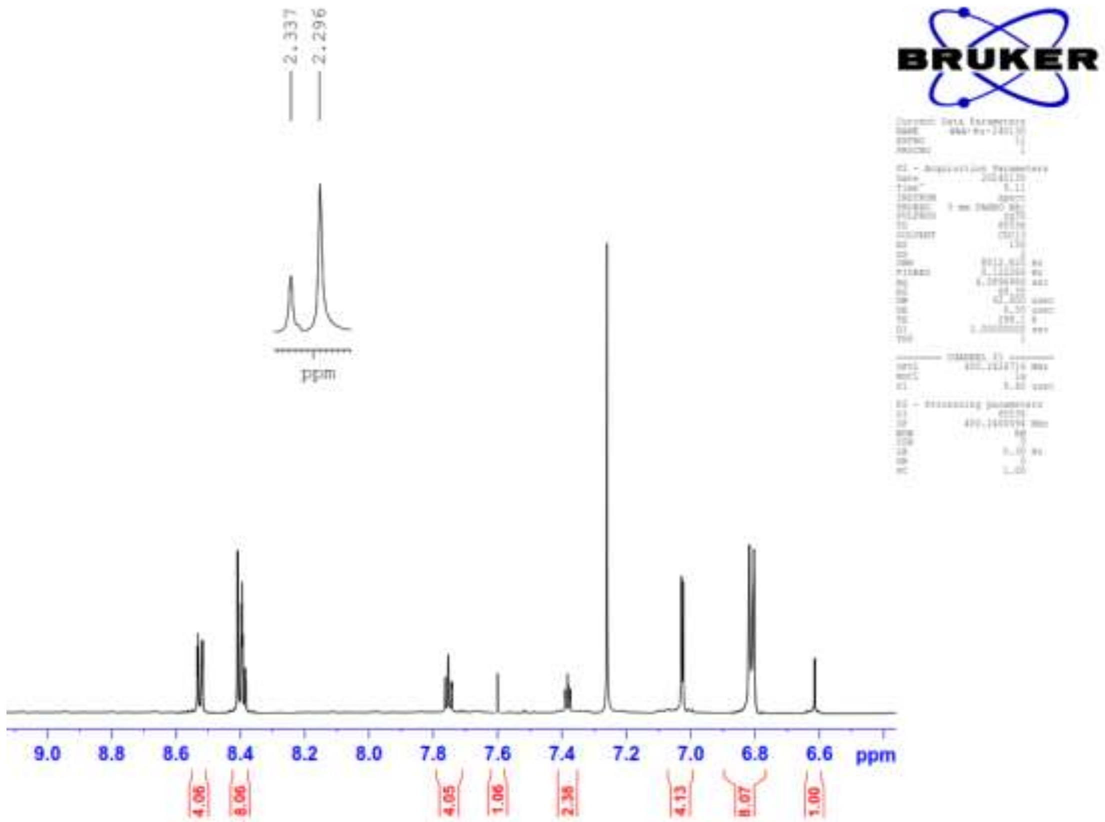

Fig. S4. ^{1}H NMR ($CDCl_3$) of Ru complex (**4**)

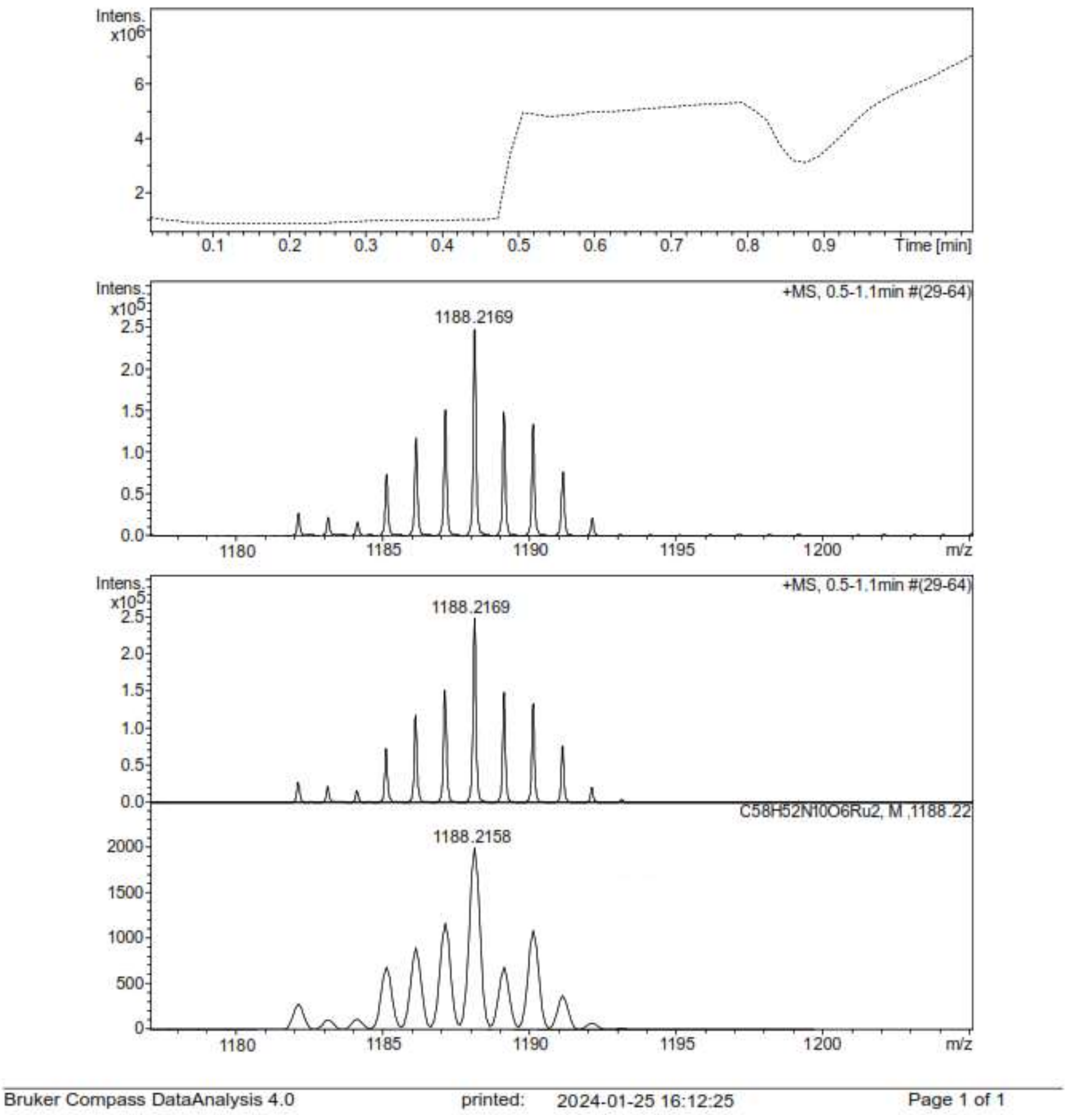

Fig. S5. HRMS of Ru complex (**4**)
